# Evaluation of the efficacy of entomopathogenic nematodes on *Ctenocephalides felis felis* larvae (Siphonaptera: Pulicidae)

**DOI:** 10.1590/S1984-29612024027

**Published:** 2024-06-17

**Authors:** Ana Caroline Ferreira de Souza, Danielle Pereira da Silva, Américo de Castro Monteiro, Vânia Rita Elias Pinheiro Bittencourt, Avelino José Bittencourt, Thaís Ribeiro Correia, Melissa Carvalho Machado do Couto Chambarelli

**Affiliations:** 1 Programa de Pós-graduação em Ciências Veterinárias, Universidade Federal Rural do Rio de Janeiro – UFRRJ, Seropédica, RJ, Brasil; 2 Departamento de Parasitologia Animal, Universidade Federal Rural do Rio de Janeiro – UFRRJ, Seropédica, RJ, Brasil; 3 Departamento de Medicina e Cirurgia Veterinária, Universidade Federal Rural do Rio de Janeiro – UFRRJ, Seropédica, RJ, Brasil

**Keywords:** Heterorhabditis bacteriophora, Heterorhabditis indica, fleas, biological control, Heterorhabditis bacteriophora, Heterorhabditis indica, pulgas, controle biológico

## Abstract

*Ctenocephalides felis felis* is a relevant and widely distributed ectoparasite that acts as a vector of disease-causing pathogens. Moreover, it is responsible for economic losses due the use of harmful chemicals to the environment and that favor the emergence of insecticide-resistant populations. Research on entomopathogenic nematodes may open up an alternative route to the insect chemical control. The present study aimed to evaluate the killing efficacy of *Heterorhabditis bacteriophora* (HP88) against *C. felis* larvae in 400 μL, 600 μL and 1000 μL of suspension containing 120, 160 and 200 infective juveniles/larva and 600 μL of suspension containing the same concentrations of *Heterorhabditis indica* (LPP30), divided into two groups (absence and presence of diet) and a control group with three replications containing only distilled water. In the bioassay with *H. bacteriophora*, the groups in 600 μL of suspension showed higher mortality rates than those in the other tested volumes, which were above 80% at all concentrations. On the other hand, *H. indica* achieved mortality rates above 70% at all concentrations used. Results indicate that flea larvae are susceptible to *in vitro* infection by *H. bacteriophora* and *H. indica*.

## Resumo

*Ctenocephalides felis felis* é um ectoparasito de relevância, ampla distribuição geográfica, vetor de patógenos causadores de doenças e responsável por perdas econômicas relacionadas ao uso de produtos químicos que causam impacto ambiental e aparecimento de populações resistentes. Pesquisas com nematoides entomopatogênicos podem ser uma alternativa ao controle químico desse inseto. O presente estudo objetivou avaliar a eficácia de *Heterorhabiditis bacteriophora* (HP88) em larvas de *C. felis* em 400 μL, 600 μL e 1000 μL de suspensão contendo 120, 160 e 200 juvenis infectantes/larva e 600 μL de suspensão contendo as mesmas concentrações de *Heterorhabditis indica* (LPP30), divididos em dois grupos (ausência e presença de dieta) e grupo controle com três repetições contendo apenas com água destilada. No bioensaio com *H. bacteriophora* observou-se que os grupos com 600 μL de suspensão apresentaram melhores percentuais de mortalidade em relação aos outros volumes testados, sendo estas acima de 80% para as três concentrações. Já *H. indica* apresentou percentuais de mortalidades acima de 70% para as três concentrações utilizadas. Os resultados indicam que as larvas de pulga são suscetíveis a infecção *in vitro* por *H. bacteriophora* e *H. indica*.

## Introduction

Fleas are small, apterous, ectoparasitic, hematophagous insects belonging to the order Siphonaptera (Linardi, 2017), which play an important role in veterinary medicine and public health, acting as vectors of several pathogens, including bacteria such as rickettsia that causes typhus, helminths and protozoa (Dryden & Rust, 1994; Linardi & Guimarães, 2000; Rust, 2005). These insects can be controlled mechanically and chemically, the latter being the prevailing route, which involves interfering in the parasite’s biological cycle (Rust, 2005). *Ctenocephalides felis* (Bouche) has proved to be resistant to common insecticides, especially carbamates, organophosphates and pyrethroids (Rust, 2020).

Entomopathogenic nematodes (EPNs) are organisms that can cause pathogenesis and mortality in arthropods and can potentially be used in the biological control of insect pests, especially those that have a stage of development in the soil (Dolinski & Moino, 2006). EPNs have symbiotic bacteria in their digestive tract, which are released into the insect’s body after the penetration of infective juveniles (IJs), leading to rapid death (24 to 48 hours) of the host due to septicemia (Dowds & Peters, 2002). Current research describes the ability of EPNs to infect and kill arthropods of veterinary importance, providing a new alternative for the control of these pests, such as ticks (Monteiro et al., 2020), mosquitoes (Cardoso et al., 2015), flies (Leal et al., 2017; Monteiro Sobrinho et al., 2021) and mollusks (Amaral et al., 2021). As for fleas, Silverman et al. (1982) evaluated the susceptibility of *C. felis* larvae to entomopathogenic nematodes of the species *Steinernema carpocapsae* Weiser (Syn. *Neoplectana carpocapsae* Nematoda: Steinernematidae), while Samish et al. (2020) evaluated the efficiency of *Heterorhabditis bacteriophora* Poinar, (Nematoda: Heterorhabditidae), *S. carpocapsae* Weiser*, Steinernema feltiae* Filipjev (Nematoda: Steinernematidae), and *Steinernema riobrave* Cabanillas, Poinar and Rauston (Nematoda: Steinernematidae), in association with entomopathogenic fungi in the control of flea larvae.

The purpose of this study was to make an *in vitro* evaluation of the killing efficacy of *H. bacteriophora* (HP88) and *H. indica* (LPP30) on seven-day-old larvae of *C. felis*, using different concentrations of nematodes and in the presence or absence of substrate for the development of flea larval stages.

## Materials and Methods

The 7-day-old *C. felis* larvae used here were obtained from a flea colony maintained for research at the same institution where the experiments were carried. The eggs were kept on a diet necessary for larval development containing wheat bran, washed sand and dehydrated animal blood according to the methodology of Vieira et al. (2010) and after 7 days of hatching, the larvae were collected for infections.

The EPNs used in this study were reproduced *in vivo* in larvae of *Galleria mellonella* Linnaeus (Lepidoptera; Pyralidae), following the method described by Lindegren et al. (1993). After being contaminated by the infective juveniles (IJs), the *G. mellonella* larvae were incubated for seven days in a climate-controlled BOD incubator with controlled temperature (Eletrolab®, model EL 202/4) at 25±1ºC, 80 RH. These larvae were then placed in a White trap (White, 1927). The newly produced IJs were collected for five days and stored in 40 mL cell culture flasks containing autoclaved distilled water and kept in the aforementioned incubator (Eletrolab®, model EL 202/4) at 16±1°C, 80 RH. At the end of the fifth day of collection, the IJs were counted for use in the infection of seven-day-old *C. felis* larvae. The infective juveniles were quantified by examining 12 aliquots of 10 μL containing the IJs under a bright field optical microscope (10x objective lens).

The highest and lowest number of IJs observed were discarded, and a simple average of the remaining values was calculated to determine the EPN concentration within each vial (Taylor et al., 1998). The suspensions obtained were adjusted to the three concentrations of EPNs used in the biological assays described below.

The experiment was divided into two groups, one with the absence and the othe with the presence of a specific diet for flea larvae to verify possible interference in the nematodes' ability to reach and penetrate the host, in addition to avoiding the occurrence of cannibalism among the larvae in case of food shortages.

The mortality rate (MR) was estimated based on the ratio of the number of dead

*C. felis* larvae to the total number of larvae used. After determining the mortality rates achieved in each treatment, the corrected mortality rate (CMR) was estimated using Abbott’s formula (Abbott, 1925), described below.


$$CMR\left(\%\right)\;=\;\left(\%Mo\;-\;\%Mb\;/100\;-\;\%Mb\;\right).\;100$$ (1)

Mc = corrected Mortality; Mo = observed Mortality;

Mb = Mortality of blank (control group)

All the factors (amount of solution used, presence of diet, EPN concentration) were evaluated separately, as were the interactions between them. A statistical analysis was carried out using SISVAR statistical software (Ferreira, 2011). The mortality data were subjected to the Shapiro-Wilk normality test and, having determined the normality of the data, they were subjected to an analysis of variance (ANOVA), followed by the F test with 1% significance (p≤0.01) or the Tukey test with a 5% level of significance (p≤0.05). For statistical purposes, the flea larvae used for dissection in both bioassays were considered dead, since they showed no motility, color changes, or EPNs inside them.

### First bioassay

The first bioassay consisted of three different treatments using three concentrations of *H. bacteriophora* HP88 IJs (A: 120; B: 160 and C: 200 IJs/flea larva) in three volumes of suspensions (400 μL, 600 μL and 1000 μL), each divided into two groups, one containing a diet to maintain flea larvae, and the other without a diet. Each treatment, which was repeated three times, was performed on a Petri dish (6cm diameter) containing previously sterilized filter paper and 10 seven-day-old *C. felis* larvae. An experimental control group was also set up, using only distilled water on filter paper (blank), to which were added 10 seven-day-old flea larvae. The bioassay was performed in a BOD incubator at 25±1ºC, 80 RH, and examined daily for a period of 48 hours.

After the observation period, White traps (1927) were set up to recover IJs of *H. bacteriophora* HP88 in order to infect *G. mellonella* and confirm the possible maintenance of the EPN’s biological cycle.

The mortality rate of *C. felis* larvae was assessed based on observations of characteristics such as larval mobility, change in color and the presence of juvenile and adult nematodes inside the flea larvae.

### Second bioassay

In this assay, *H. indica* (LPP30) was used in the same quantities as those in the first bioassay (A: 120; B: 160 and C: 200 IJs/flea larva), but in only 600 μL of suspension; the bioassay was divided into two groups, one with a specific diet for flea larvae and the other group without a diet. The experiment was carried out for a period of 48 hours in a BOD incubator at 25±1ºC, 80 RH, and was examined daily. After the first 24 hours, one larva was removed from each Petri dish, dissected and examined under a stereoscopic microscope to determine whether it had been infected by EPNs. After 48 hours, White (1927) traps were set up to recover IJs. The recovered infective juveniles were used to infect *G. mellonella* in order to determine whether the IJs could infect them. As occurred in the first bioassay, the mortality rate of *C. felis* larvae was assessed based on characteristics such as larval mobility, color alterations, and the presence of nematodes inside flea larvae.

## Results

The first bioassay performed with *H. bacteriophora* (HP88) in 1000 µL of suspension resulted in 100% mortality of *C. felis* larvae within 24 hours in all the treatments, including the control group. The flea larvae did not change color and no EPNs were detected inside them, indicating that there was no infection by IJs and no mortality caused by the symbiotic bacteria. This suggests that larval mortality may have been caused by excess de humidity on the Petri dish. No statistical evaluation was made due to the death of all the flea larvae within the first 24 hours, including the controls.

**Figure 1:**
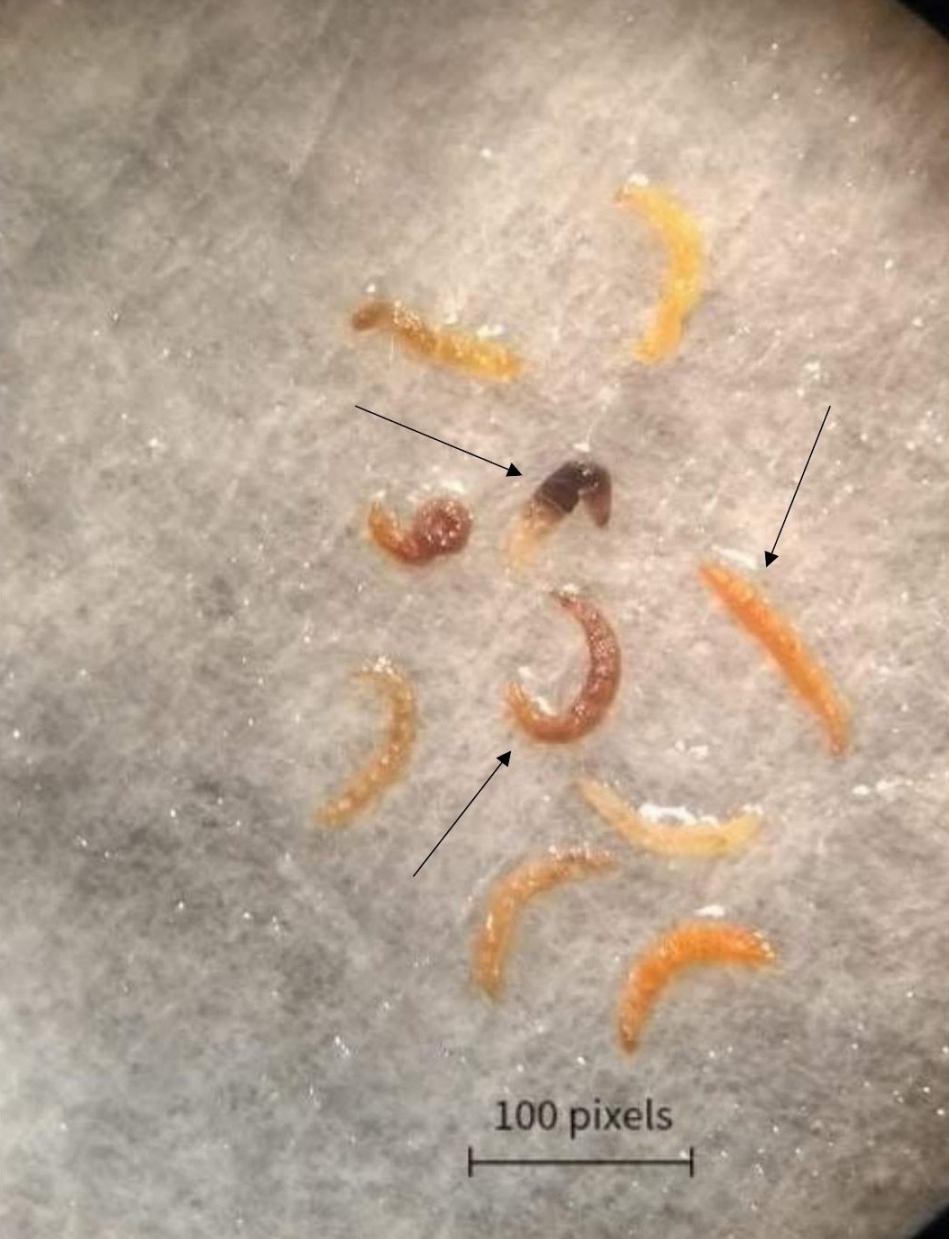
Arrow indicating *Ctenocephalides felis felis* larvae color change after infection with the entomopathogenic nematode *Heterorhabditis bacteriophora* (HP88). (Source: Personal file). Labscope 100 pixels=26,458mm.

An evaluation of the biological assays containing *H. bacteriophora* (HP88) in 400 µL and 600 µL of suspension indicated that some larvae showed altered mobility and color after infection (Figure 1). The mortality caused by IJs was confirmed through the dissection of flea larvae, which revealed the presence of juvenile and adult forms of EPNs inside them. However, the mortality rate of flea larvae was calculated 48 hours after infection with IJs, which again indicated the presence of juveniles and adults inside the flea larvae. The mortality rate achieved varied according to the volume of suspension used.

The data obtained in biological assays containing 400 µL and 600 µL of suspension of *H. bacteriophora* IJs were evaluated by an analysis of variance (ANOVA), which indicated that the nematode concentrations (EPNs) and the amount of suspension used produced significant effects.

An overall evaluation, regardless of the amount of suspension, indicated that all the EPN concentrations resulted in a increase in the mortality of flea larvae when compared to the control, albeit without statistically significant differences between the concentrations of EPNs used.

A separate analysis revealed that the use of 400 μL of suspension led to a lower mortality rate of flea larvae when compared to 600 μL, and that these suspension volumes resulted in mortality rates of 51.3% and 69.7%, respectively. However, an analysis of the effect of the presence or absence of diet indicated that this factor did not influence larvae mortality rates, and no significant differences were found between them.

An analysis of the mortality of flea larvae in 600 μL of suspension indicated that the presence of IJs of *H. bacteriophora* (HP88) increased their mortality rate (Table 1), regardless of the quantity of EPNs, without statistical differences between nematode concentrations (120, 160 and 200 EPNs/larva). A comparison of the presence or absence of diet resulted in the same finding. However, the control groups showed no significant mortality rates.

**Table 1:** Mean mortality rates of *Ctenocephalides felis felis* larvae infected with *Heterorhabditis bacteriophora* (HP88) in 600 μL of suspension.

Mortality rate (%) of *Ctenocephalides felis felis* larvae in 600 µL of suspension containing entomopathogenic nematodes *Heterorhabditis bacteriophora* (HP88)				
Treatments	0 EPNs	1200 EPNs	1600 EPNs	2000 EPNs
With diet	16.6 Bb	100.0 Aa	83.3 Aa	83.3 Aa
Without diet	6.6 Bb	92.6 Aa	90.0 Aa	85.2 Aa
Mean	11.6 Bb	96.3 Aa	86.6 Aa	85.7 Aa

Means followed by the same uppercase letters in the rows and lowercase letters in the columns do not differ from each other according to the Tukey test (p≤0.05).

Larvae from the experimental group containing 1000 µL of suspension were discarded. Extensive desiccation of flea larvae occurred in treatments using 400 µL of suspension of *H. bacteriophora* (HP88) EPNs, which prevented the recovery of *H. bacteriophora* IJs in White traps (1927). The White trap (1927) set up with the larvae of the biological assay containing 600 µL of suspension captured a variable number of IJs that were used for infecting *G. mellonella*, which was successful. This demonstrates that the infection of flea larvae successfully generated entomopathogenic nematodes that were able to complete their cycle and infect other arthropod species.

**Figure 2:**
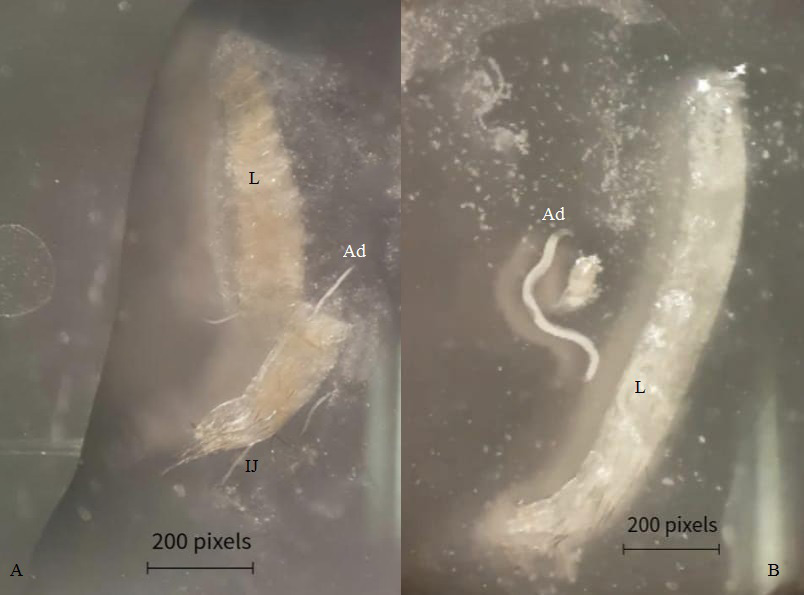
(A) *Ctenocephalides felis felis* larvae (L) infected with *Heterorhabditis indica* adults (Ad) and infective juveniles (IJ) revealed by dissection (Source: Personal files); (B) *Ctenocephalides felis felis* larvae (L) infected with *Heterorhabditis indica* adults (Ad). Labscope 200 Pixel = 52916.6 μm.

The analysis of the second bioassay revealed non-motile flea larvae with altered coloration, which, when dissected, showed the presence of EPN juveniles and adults inside (Figure 2 A and 2B).

Data relating to the mortality of *C. felis* larvae by the nematode *H. indica* (LPP30) were evaluated using ANOVA, which indicated that the EPN concentration significantly affected the mortality rate of flea larvae when compared with the control groups. However, the mortality rates of the diet and non-diet groups showed no statistically significant differences (Table 2).

**Table 2:** Average mortality rate of *Ctenocephalides felis felis* larvae as a function of *Heterorhabditis indica* (LPP30) nematode concentrations and the use of a specific flea diet.

Mortality rate (%) of *Ctenocephalides felis felis* larvae in 600 µL of suspension containing entomopathogenic nematodes *Heterorhabditis indica* (LPP30)				
Treatments	0 EPNs	1200 EPNs	1600 EPNs	2000 EPNs
With diet	10.0 Bb	85.2 Aa	75.0 Aa	80.00 Aa
Without diet	6.7 Bb	81.4 Aa	74.0 Aa	93.33 Aa
Mean	8.3 Bb	83.3 Aa	74.5 Aa	86.7 Aa

Means followed by the same uppercase letters in the rows and lowercase letters in the columns do not differ from each other according to the Tukey test (p≤0.05).

The use of White traps (1927) enabled us to observe the emergence of IJs of *H. indica* (LPP30) that were used to infect *G. mellonella*, demonstrating that this species was able to complete its cycle in flea larvae and generate new infective juveniles.

## Discussion

There are few reports in the literature about the infection of *C. felis* fleas with EPNs, and most of those reports describe infection of these fleas with nematodes of the genus *Steinernema* and the species *H. bacteriophora* (HP88). Therefore, this is a pioneering study which involved an evaluation of the infection of flea larvae with *H. indica* (LPP30) EPNs.

Silverman et al. (1982), who evaluated the pathogenicity of *S. carpocapsae* in immature stages of *C. felis*, detected the presence of adult EPNs in the insect’s hemocoel 48 hours after infection. In the present study, although a different species of EPNs (HP88) was used, it was found that 24 hours after exposure some flea larvae already contained EPNs inside them, and after 48 hours most of the flea larvae contained adult EPNs. Hence, our findings corroborate those presented by Silverman et al. (1982).

In their experiment, Silverman et al. (1982) did not detect the presence of infective juveniles of EPNs. Their results differ from those of our study, in which White traps (1927) were set up to capture infective juveniles (IJs) that had been used in the infection of *G. mellonella*, continuing the reproductive cycle of EPNs and thus demonstrating that after infecting flea larvae, it is possible to obtain infective juveniles.

Silverman et al. (1982) reported a mortality rate of up to 90% of flea larvae. However, the results of those authors were inferior to those of this study, in which a higher percentage of infection with *H. bacteriophora* (HP88) was achieved at a concentration of 120 IJs/larva than that obtained by Silverman et al. (1982), and a mortality rate varying from 74% to 100%. This suggests that the EPN species evaluated here, *H. bacteriophora* and *H. indica*, are more efficient than those previously studied by the aforementioned researchers, a fact that may have been favored by the morphology of the EPNs of the genus *Heterorhabditis*, since these EPNs are smaller and have minuscule appendages in the cephalic region (chitinous teeth) that enable them to actively penetrate the insect’s cuticle (Forst & Clarke, 2002).

It is known that infective juveniles require moisture for their survival and mobility, without which they can become desiccated (Rohde et al., 2010). The average mortality rate achieved in the biological assay using 400 μL of suspension was lower than that attained with 600 μL of suspension. This corroborates the results reported by Samish et al. (2020), who evaluated five strains of EPNs in different situations, and found that they caused varying mortality rates of flea larvae. These researchers stated that changes in temperature and relative humidity (RH) affected the efficacy of all the nematode strains, and that raising the temperature and humidity increased the mortality rate of flea larvae. The same finding regarding humidity was observed in studies carried out by Silverman et al. (1982), who enhanced flea larvae mortality rates by increasing relative humidity from 7% to 22%.

Humidity is important for both EPN and *C. felis* larvae, as they require moisture to survive. However, excessive moisture can cause mortality of flea larvae, as was observed in a biological test using 1000 μL of suspension, which caused a 100% mortality rate of larvae, including the control groups, thus confirming the reports of Dryden & Rust (1994) and Silva et al. (2008).

With regard to substrate, Arriaga & Cortez‐Madrigal (2018) report that the use of substrate containing a high percentage of organic matter could interfere with the survival of EPNs, favoring the development of microorganisms that can reduce oxygen availability. Monteiro Sobrinho et al. (2016) reported the same situation, stating that EPN infectivity declined as a result of using filter cake as a substrate for the development of *Stomoxys calcitrans* Linnaeus (Diptera: Muscidae) larvae. The use of this substrate led to lower mortality rates than those reported by Leal et al. (2017), who used EPNs to control stable flies, albeit without utilizing any substrate. However, this problem was not encountered in the present study, since the presence of a diet for flea larvae did not interfere in the larvae mortality rate when compared that of larvae reared without a diet. In other words, even in the presence of substrate to aid in the development of the EPNs’ target insect larvae, the moisture level sufficed to ensure the mobility and survival of the juveniles.

*Ctenocephalides felis* larvae cannibalize each other if they do not find food (Dryden & Rust, 1994). This behavior was observed in the control treatment of the experimental group raised without a diet, using 400μL of solution. This may be attributed to the low moisture content of the filter paper, which may have hindered the mobility and penetration of the infective juveniles into the flea larvae, enabling the latter to remain alive but without food for a longer period of time, causing them to eat each other.

## Conclusions

The present study demonstrated that infections of 7-day-old *C. felis* larvae with entomopathogenic nematodes of the species *H. Bacteriophora* (HP88) and *H. Indica* (LPP30) under laboratory conditions were well succeeded. The results demonstrated that *C. felis* larvae are susceptible to infection by both species of entomopathogenic nematodes evaluated and the mortality rate will depend on the EPNs species used and the umidit of the environment. The data presented in this study, especially regarding to *H. Indica* which is the first report of its use in *C. felis* larvae, are important because it shows the susceptibility and mortality of the flea larvae to the EPNs and the capacity of these species of nematodes to make at least one complete life cycle inside the insect host. This is the first step towards developing biological control strategies where EPNs can be used as a tool to control *C. felis*.
